# Quantifying life: Understanding the history of Quality-Adjusted Life-Years (QALYs)

**DOI:** 10.1016/j.socscimed.2018.07.004

**Published:** 2018-08

**Authors:** Eleanor MacKillop, Sally Sheard

**Affiliations:** Department of Public Health and Policy, Institute of Psychology, Health and Society, University of Liverpool, UK

**Keywords:** United Kingdom, QALY, History, Health policy, Health economics, Multiple-streams analysis

## Abstract

Quality-Adjusted Life-Years (QALYs) are central to healthcare decision-making in Britain and abroad, yet their history is poorly understood. In this paper, we argue that a more in-depth and political history of the QALY is needed to allow a critical evaluation of its current dominance. Exploiting rich data from archives and 44 semi-structured interviews conducted between 2015 and 2018, we employ Multiple Streams Analysis to construct a complex and dynamic picture of how the idea of QALYs emerged and was adopted within UK health policy. Through its historical and political approach, the paper illuminates the relative roles in the policy-making process of experts (especially economists) and politicians as ‘entrepreneurs’ in the development of new ideas; how these were influenced by negotiation within established and emerging institutional structures; and the role of serendipity and crisis.

## Highlights

• Constructs a first detailed history of the Quality-Adjusted Life-Years (QALY) index.
• Combines history and political science methodologies to explain health policy-making.
• Demonstrates the role of individuals, networks and serendipity in the emergence of new health policies.

## 1 Introduction

The Quality-Adjusted Life-Years (QALYs) measurement – a tool developed to evaluate the cost-effectiveness of treatments – is central to healthcare decision-making in Britain – where it forms the basis of the work of the National Institute for Health and Care Excellence (NICE) – and in many other countries. The first use of the term ‘QALY’ is contested (Cohen, 1998). In the US and Canada in the late 1960s, the operational research (OR) community contributed to debates on Quality-of-Life (QoL) questions. Klarman et al. (1968) published a key article articulating the idea of the QALY whilst Bush et al. (1972) were the first to use the term ‘QALY’ (see also Weinstein and Stason, 1977; Zeckhauser and Shephard, 1976). There were also seminal contributions from the evidence-based medicine group at McMaster led by Torrance et al. (1972).

There are multiple definitions of QALYs. This paper uses NICE's definition of “[a] measure of the state of health of a person or group in which the benefits, in terms of length of life, are adjusted to reflect the quality of life” (NICE, 2016). QALYs bring together morbidity and mortality in a single ratio to evaluate the outcome of health interventions. It is variously referred to as an ‘index’, ‘tool’ or ‘measurement’. Although it is now a widespread concept, the history of the QALY remains obscured and/or often simplified. We suggest that this is due to two factors: previous histories have been written by non-historians, and they have not exploited archival source analysis or oral history.

What is needed is a more rigorous history of the QALY, emphasising the political economy of its emergence and subsequent adoption within health policy and decision-making. This is necessary to support debates on whether QALYs are the best available tools for decision-making and how they might contribute to, or hinder, the goal of equity in healthcare (Knapp, 2015). If the current and future use of QALYs is to be effectively analysed, it is vital to understand how and why they became so essential to policy-makers and health service professionals. This paper blends conventional historical research methodologies – archival analysis and oral history – with political science. We use Kingdon's (1984; 1993) Multiple Streams Analysis (MSA) which enables the construction of complex and dynamic explanations of how policy ideas ‘catch on’, combining notions of policy entrepreneurs, streams, and negotiation/bargaining.

The paper is organised as follows: we review the available literature on the history of the QALY and propose a framework highlighting the politics and wider context of its formulation and implementation. The research methods are discussed, before we construct a history of the QALY by articulating Kingdon's streams. We conclude by discussing the importance of critical historical research for effective health policy development.

## 2 Developing a critical history of QALYs

Our initial literature review on the history of QALYs identified remarkably few sources, most of which had been written by economists. These sources tend to present a partial history in which there is little discussion of the interaction between research and policy (Hurst, 1998; Kind, 2005; Williams, 2005). Some offer good historical accounts but often from one person's view (Rosser, 1993; Williams, 2005; also Ryan and Gerard, 2014 on Mooney's contribution). Other sources have focused on the contributions of disciplines other than health economics, QALY theory (Coast, 2018; Forget, 2004; Gold et al., 2002), or its application in specialised areas such as renal dialysis (Cohen, 1998; Holland, 1991; Stanton, 1999). Medical sociologists provide a useful history of QoL measures and their social dimension but here again there is no in-depth discussion of the policy context (Armstrong et al., 2007). Some develop detailed accounts but lack a political or policy dimension (Ashmore et al., 1989; Blaug 1998; Harris, 1987; Nord, 2001; Williams, 1985b). The most interesting sociological approach is probably Wahlberg and Rose's international analysis of a range of quality of life measurements (Wahlberg and Rose, 2015).

Our analysis combines political theory with history. Historians have only recently begun to investigate the potential of such methodologies for their traditional materials, but it is promising (Gorsky and Millward, 2017). For this case study, we found Kingdon's Multiple Streams Analysis (MSA) (1984) particularly relevant for examining the complex QALY history. It posits three different ‘streams’ – policy, politics, and problems – where ideas float, and which merge together to open a window of opportunity, through the negotiating work of policy entrepreneurs.

**Fig. 1:**
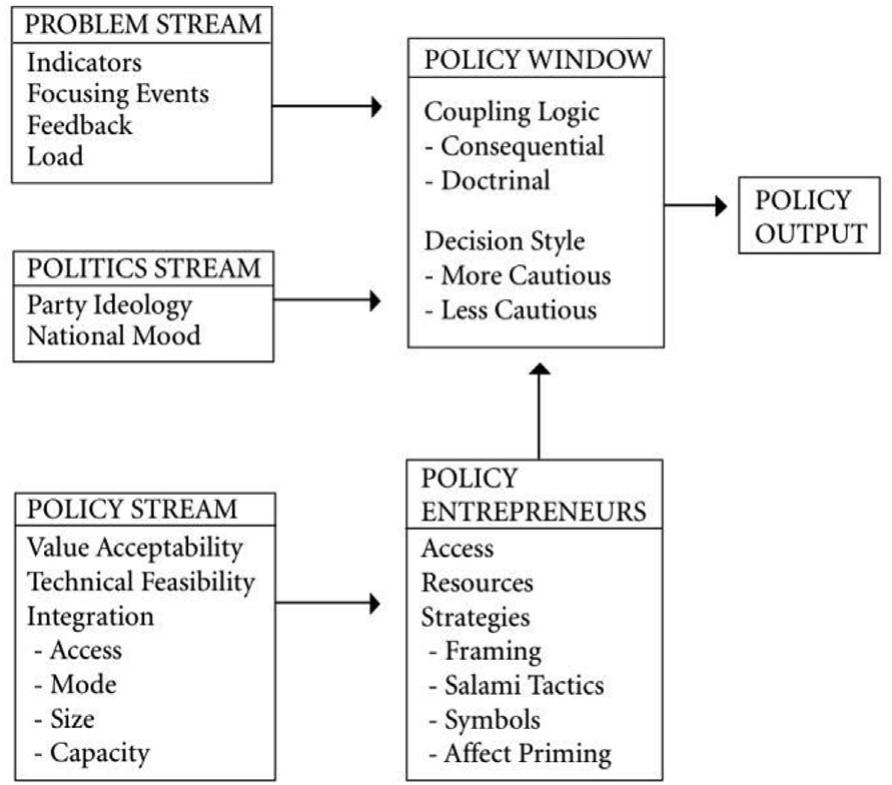
Multiple Streams Analysis schema (based on Zahariadis, 2014).

The *policy stream* (in Fig. 1) is where policy ideas are formulated within what Kingdon calls a competitive *primeval soup* where ideas compete and the ‘fittest’ survive. Ideas ‘float’ within policy communities and networks composed of think-tanks, academic centres, civil servants, political parties or Select Committees. The survival of ideas depends on three criteria: value acceptability, technical feasibility, and network integration (ideas will be debated in different networks and communities with different levels of competition and openness impacting on the potential growth of the idea). The *problem stream* is where issues emerge, based on indicators such as low economic growth or rising costs of care, and focusing events which catch popular attention. It also responds to ‘load effect’, in which policy innovation is a response to the multiplication or heightening of several problems. The *politics stream* is where individuals such as politicians and civil servants evaluate the national mood and pressure groups' support for an idea, based on the contemporary political context, including factors such as recent elections, arrival of newly elected politicians and opinion polls.

Kingdon also describes the roles of individuals or institutions as *policy entrepreneurs,* or “surfers waiting for the right wave” (Kingdon, 1984, p.173). Entrepreneurs influence policy-making by bringing together the three streams around a specific issue. Entrepreneurs may be found in all three streams, and often move between these streams. Once the streams have been coupled by entrepreneurs, a brief and unpredictable *window of opportunity* opens, “for advocates of proposals to push their pet solutions, or to push attention to their special problems” (Kingdon, 1993). These political science analytical frameworks are not routinely used within health services research: only three articles were found specifically articulating MSA for a health problem (McMillin, 2014; Shapiro et al., 2017; Whiteford et al., 2016). However, MSA presents clear advantages for understanding complex cases of intertwined interests and individuals such as we see with QALYs. We critically applied MSA to make sense of individuals, ideas and wider phenomena, iteratively applying concepts alongside the rich data collected to achieve the best possible explanation (Glynos and Howarth, 2007). Rather than these streams being integrated into how interview questions were formulated, the process was more iterative, with questions focusing on a number of issues around the emergence, growth and influence of health economics. Only during the analysis did the theme of QALYs and the potential for analysing this specific issue through MSA emerged. Then followed a dialogue between the theory and the empirical data to develop the best possible explanation for the emergence of QALYs.

## 3 Methods

We collected archival material from various sources, including the National Archives (TNA), uncatalogued Department of Health files (80 from TNA; 50 files from DH Burnley) and from University of York archives. Ethical approval guaranteeing confidentiality and data protection was obtained to conduct semi-structured oral history interviews with 44 participants, mostly UK-based retired and practising academics and civil servants. These interviews were recorded digitally, fully transcribed and, where requested, anonymised. These are referred to as civil servants (CS and number) and academics (A and number). Participants were asked about their understanding of the emergence of QALYs, the role played by Government and possible alternatives. To gauge the awareness of the British medical profession in the discipline of health economics, British Medical Journal (BMJ) papers between 1930 and 1990 were searched using the keyword ‘health economics’ (n = 385) via access to a trial text mining project based at the University of Manchester. For the specific concept of QALYs, the BMJ and the Lancet were searched for the period 1960 and 1999 (n = 345). This demonstrated first uses of the term QALY from 1984 (BMJ) and 1985 (Lancet). Secondary literature on the development of health economics was also collected (Croxson, 1998; Hurst, 1998; Smee, 2005). These data were coded in NVivo 11, using broad themes such QoL, creation, actors, relations, resistance, and competition from other disciplines/groups. These themes, and later codes, were adapted as each source was re-read.

## 4 The formulation of the QALY concept in the UK

Here, we construct a history of the QALY by characterising the three streams.

### 4.1 The problem stream

The British National Health Service, founded in 1948, is a universal system in which healthcare is free at the point of delivery and funded through central government taxation. Historically, until the creation of the NHS Management Executive in 1989, it was managed via the Ministry of Health, later replaced by the Department of Health and Social Security (DHSS) in 1968, and the Department of Health (DH) in 1988. There has always been political accountability for the NHS in Parliament through the Secretary of State for Health, who is advised by permanent, non-political civil servants, and, since the late 1960s, by temporary special advisers.

In the late 1960s and early 1970s, the UK experienced economic shocks, including the international OPEC oil crisis. The cost of healthcare increased because of a number of factors. These included the expense of innovations in health technology such as organ transplants and renal dialysis, and the impact of an ageing population, who had more healthcare needs. Between 1960 and 1982, average real-term spending on healthcare ranged from 3.9% to 5.9% of GDP, with two clear increases during the mid-1970s (Health Foundation, 2015). There were also raised expectations about the quality and availability of care for what may have been seen as relatively insignificant needs in the early years of the NHS (Smith, 1987). One handwritten DHSS memorandum from 1970 remarked that “the more people we keep alive, the more it will cost the service” (TNA, BN13.197). The concerns about the viability of the NHS have been present from its creation and regularly surfaced through investigations such as the Guillebaud Committee on the cost of the NHS (1953–1956) and the Hinchcliffe Committee on the cost of prescribing (1957–1959). There was however a clear increase in attention from the late 1960s, stimulating studies on efficiency and cost-effectiveness. The first major reorganisation of the NHS in 1974 was one result of this chronic concern. However, it did not solve the issue, and a series of crises, including staff strikes and lengthening waiting lists contributed to a sense of ‘problem’. Market solutions such as willingness-to-pay or demand versus cost were not appropriate in this universal, state provided system. A modified form of ‘rationing’ was introduced through the economics-inspired Resource Allocation Working Party (RAWP) formula in 1976, which attempted to redistribute the budget to areas with greater need.

Furthermore the ‘machine’ of government was increasingly subject to scrutiny, especially the issue of efficiency and value for money. The Plowden and Fulton reports (1961 and 1968 respectively) proposed a greater role for research and expertise in policy-making. The Treasury and the DHSS demanded greater accountability for outputs. However measuring outputs in health was notoriously difficult compared to areas such as defence or transport (Colvin, 1985). Kingdon's ‘load effect’ was emerging, as multiple problems were layered on top of each other. For Rudolf Klein, these crises and reforms “transformed the language in which every issue was debated” within the NHS. Now economics was enabling “non-numerate people like me to talk about issues like cost-benefit analysis” (Interview, 2016).

There were specific ’focusing events' that helped economists to articulate problems. Alan Williams and Alan Maynard, pioneering academic health economists at the University of York, used stories of patients generating huge media coverage to highlight the inadequate setting of priorities in the NHS. In 1986, Maynard drew attention to the considerable sum of money spent on new-born babies in intensive care, while thousands of (usually elderly) patients were awaiting cheaper interventions such as hip replacements (Ashmore et al., 1989, pp.60–81). Economists offered “a more standardised approach of looking at cost-effectiveness analysis” (CS7). However, an analytical tool was not available to assist with making such choices:

there were questions like the DH could stop doing routine screenings for TB, but the DH didn't have a mechanism like NICE to recommend to the NHS that they should do certain things. (A3)

### 4.2 The policy stream

Quality of Life (QoL) – refers to philosophical, medical and sociological debates over whether and how to attribute different values to different lives and different health states. Measurements of QoL became a research focus in the clinical/medical community during the 1940s and 1950s (Cohen, 1998; Costanza et al., 2007). Medical professionals combined morbidity and mortality data into tools such as the Nottingham Health Profile and the Karnofsky index (Gudex, 1986). International organisations such as the World Health Organisation (WHO) were also interested in QoL from the perspective of population health measurement, which traditionally relied on population surveys (WHO, 1957; Sheard, 2013). Other communities such as academic philosophers (Harris, 1987; Koch, 2000), OR, evidence-based medicine (Torrance et al., 1972) and transport economics (Jones-Lee, 1976) were interested in the QoL question (cf. MacKillop, 2017).

Earlier in the 1960s, researchers had become interested in standardising the description of individuals' health states and scoring them in order to rank them on a scale of severity (Katz et al., 1963), although some studies moved towards devising comprehensive models based on mathematics, such as those combining morbidity and mortality (Chiang, 1965). From the late 1960s, a final key move was made towards cardinal measurements to order states of illness as well as quantify their undesirability (see Rosser, 1983).

In parallel to the North American research mentioned above, British researchers had also been active in the 1960s in devising outcome measurement in health. During his secondment to the Treasury in 1966–1968, Alan Williams authored a paper for the Centre of Economic Studies on outcome measurement (A3). In 1971, three economists at the University of York – Tony Culyer, Bob Lavers and Alan Williams – had described the idea combining painfulness and restricted activity, representing it in a diagram (TNA, MH166/927). This paper was cited by several of our interviewees as ground-breaking (e.g. CS1; A2). One government economist noted:

Now that was a revelation to me and I remember looking at that and thinking ‘yes! That's what we're trying to do! That's outcomes!’ (Jeremy Hurst, Interview)

Archives indicate that, in the 1970s, DHSS economists began to take an interest in how other departments approached the question of “comparative cost effectiveness of life saving” (e.g. TNA, AT82/11, *Economic value of life: examination of methods of evaluating life for cost benefit analysis of road and railway safety projects*, 1 January 1971, p.2). There was disagreement among government economists over which form of valuation to adopt. Gavin Mooney, then an economist at the Department for the Environment, favoured the “Jones-Lee methodology” of valuation to measure attitudes to risk, combined with a behavioural approach, as advocated by Martin Feldstein – a US economist who had completed his PhD research on the NHS at the University of Oxford in 1967 (TNA, AT82/11, p.3). The Economic Advisers' Office was created in DHSS in 1968, with just two economists, David Pole and Jeremy Hurst, appointed in 1970 to work on health policy (there are now 54 economists working in DH, DH email exchange). Later Norman Glass joined them, working on methods to estimate NHS costs of road traffic accidents in 1975–76 (TNA, MH148/579, *Estimation of cost to the National Health Service of road traffic accidents and recuperation of costs through insurance companies: proposals, drafts, statistics and correspondence*, 1 January 1975).

Culyer, Lavers and Williams's research was limited to theory but there was a British researcher making progress in the fieldwork. Rachel Rosser was a Professor of Psychiatry at University College London/Middlesex University. From early 1970s, she developed her ‘sanative output’ measures for hospital patients, working with her husband, the operational researcher Vincent Watts. These measurements aimed at evaluating whether a hospital stay had improved a patient's health and to what extent relative to full health (Rosser and Watts, 1972). Rosser used qualitative methods for evaluating these health states such as interviewing patients and their doctors. But her research failed to attract enough attention from researchers and policy-makers to support its development into a potential policy tool.

To understand the slow maturation of QALY as an accepted outcome measurement, it is necessary to understand the growth of economics within Government, which Jeremy Hurst observed first hand (Hurst, 1998). Until 1964, when the Government Economic Service (GES) was created – a Treasury body that recruited economists for deployment across Departments –, few economists worked in Whitehall (Allan, 2008). When Wilson's Labour government was elected in 1964, only 25 full-time economists were employed. Five years later, there were just under 200 and this number doubled between 1970 and 1977. It was in this more welcoming environment that economics became increasingly present and thus its language and tools became more familiar and potentially useable to non-economist civil servants and politicians (Davis, 1998, p.5).

In the DHSS Pole and Hurst emphasised the need for more economics, the former explaining that Government economists should “do anything we can to stimulate an interest in and the use of cost-effectiveness analysis” (TNA, BN155/4, *EAO NHS: Economic Analysis*, ‘Economic appraisal’, 18 May 1972). They also advocated broadening the impact of different types of economic analysis on health policy. Pole had conducted an economic evaluation of “routine radiography to detect TB” when working at Cardiff in 1969, so when he moved to DH, “economic evaluation was on his list of things that he knew about” (A2). Economists outside DH also informed contemporary policy issues. For example, from 1971, the York-based economist Peter West conducted a study on the cost of teaching hospitals which directly informed DH policy (CS1; CS4; A2). Research on QoL measurements and other areas was enabling economists and other researchers to develop ‘usable tools’ for DH economists:

The importance of the early cooperative work on QoL measurement is that it led to a usable British and now European health outcome measure, the QALY. (Jeremy Hurst, interview)

Government economists were increasingly called upon by the ‘Top of the Office’ (senior civil servants) in DHSS to apply their analytical skills to an increasing number of topics such as providing criteria for priority setting major capital schemes in District General Hospitals (TNA, BN155.5.2, *EAO*, Meeting on morbidity indices, 4 December 1972). Thus, economic analyses, especially cost-effectiveness analysis, became embedded in DHSS.

Ministerial support was critical to securing the influence of economists in DHSS and making the QALY politically palatable. Barbara Castle (Secretary of State for Health and Social Services, 1974–1976), her junior Minister of Health, David Owen (1974–1976), and high-ranking civil servants, such as Douglas Black (Chief Scientist, 1973–1977), were enthusiastic about economics and how it could inform health (Williams, 2005). Their support was apparent to economists themselves, both within DHSS and academia:

I think the economists within the DH had a bit of a golden period. They obviously had the ear of particular ministers. Some ministers would be more interested in it than others. I think David Owen was one who was particularly interested but perhaps not the only one. I think it was about the power of the economists in DH (A2)

Yet there were some within DHSS who were not immediate converts to health economics, especially within the medical civil service. An interviewee recalled an economist who had proposed ways of measuring health outcomes being “hauled up before the Chief Medical Officer” (CS1; also reference in CS2).

Kingdon's policy stream also requires an assessment of the technical feasibility of the emerging policy idea. For the QALY, there were a number of academics struggling with developing instruments for health outcome measurement that were technically feasible. Rosser and Watts continually refined their own methodologies, moving from evaluating health states, notably distress and disability, based on legal awards for personal injuries and industrial accidents and diseases (Rosser and Watts, 1975), to collecting qualitative data from interviews with patients and health professionals and survey questionnaires to refine these health states into workable scales (Rosser and Watts, 1974; Rosser and Kind, 1978). A significant breakthrough came with the developments in Information Technology (IT) with computer scientists turned economists such as Paul Kind becoming able to collect health statuses and compare them on a much greater scale than doing so by hand. According to one interviewee involved in the development of QALYs, the biggest challenges in making the Rosser-Watts index applicable were “methodology and getting reliable data” and “talking to clinicians” about the assumptions being made by these researchers (A8), all of which required intensive research and consultation. For his part, Martin Buxton, who attempted to use an earlier form of this index in the heart transplant evaluation (Buxton, 1987), found that although it was “a useful [and] important step in the process of getting to QALYs”, it was too “crude” at the time because the weights were “not patient or population weights that one could rely on” (Martin Buxton, Interview). For several of our interviewees, making the QALY ‘usable’ in health was easier once it was a workable tool:

I think if we never had the methodology for doing these things it would have been more difficult, but I think it was easy to once you had the methodology and you found a policy hook and then there it went. (A2)

Kingdon's policy stream also requires an assessment of how the idea was integrated within distinct networks and communities. The QALY concept was weakly integrated until the 1970s, with non-academics and those outside Rosser and Watt's network of researchers unaware of their ideas. This may be linked to the absence of a network such as the Health Economists' Study Group (HESG) which, from 1972, brought together academics, government economists and others into sharing ideas and research (Croxson, 1998). Williams felt that the main drawback was that “the measurement scales were rather idiosyncratic, and difficult to interpret”, using maximum values (for perfect health) of 497, a figure which did not correspond to anything obvious (Williams, 2005, p.4).

Rosser and Williams met in the mid-1970s and began working together. In a key breakthrough in 1982, Williams proposed the integration of measures of quality of life with those of life expectancy to “capture the essence of a person's healthiness” (Williams, 2005, p.3). He also proposed the now conventional 0 to 1 scale (Kind et al., 1982). To further increase the ‘palatability’ of the idea, the “somewhat awkward term ‘sanitative output’” was dropped in favour of a simpler one: the Rosser or Rosser-Kind index (Kind, 1998, p.655).

Several applications helped to ‘soften up’ or normalise the QALY approach by demonstrating how it could help inform real NHS decision-making. We here focus on two. Williams was invited to attend a consensus development conference on Coronary Artery Bypass Grafting (CABG) organised by doctors in November 1984. He discussed “the costs and benefits of competing technologies” which, for the medical members of the panel, was “a novel challenge” (Jennett, 1985, p.717). According to Jeremy Hurst, then Senior Economic Adviser in DHSS, this was “a breakthrough moment, especially in relation to getting the acceptance of the doctors for QALYs” (Interview). In 1985, Williams (1985a) published a paper in the BMJ on CABG. This put economics into easily understood language for clinicians and demonstrated the opportunity cost of this surgical technique relative to other possible clinical interventions. Williams formulated a league table of treatments for angina, and used QALYs to rank them, using also data from Martin Buxton's recent economic analysis on heart transplant. Because there was little data available on the cost and outcomes of these interventions, the Economic Advisers' Office helped with costing, highlighting the importance of the early collaboration between DH and academia in the formulation of QALYs. Williams was asked by doctors during the conference: “how would you as an economist tell us whether we are doing too much or too little about heart surgery” (A2). He therefore included wider interventions such as bypass surgery, heart transplants and hip replacements and GP promotion of smoking cessation in the prototype QALY league table. This formed a key moment in opening a window of opportunity for QALYs, when it was combined by Williams and others with the issue of the growing cost of healthcare.

The second key development in ‘softening up’ the policy community to using QALYs came the following year. In 1986, Anne Ludbrook, a York-trained health economist who had moved to Health Economics Research Unit (HERU) at Aberdeen in 1983, was invited by the DHSS to be a member of the Forrest Commission on breast cancer screening. The Commission's report, published in 1986, was one of the first to recommend calculating the economic cost per QALY for screening for specific health conditions. According to an Economic Advisers' Office economist, until then, the cost per QALY approach was “very much a side show in the Department, even among the economists”, as the resource allocation issue took priority (CS4). The Forrest Commission was established to decide whether screening for breast cancer should be introduced for all women across the UK. As there was no useful UK data, the Commission used US and Swedish studies as well as small UK pilots. Ludbrook presented evidence in favour of screening using a life expectancy versus quality graph. The Commission was convinced, “so long as they got a number that they could use as the base estimate” (A1). However, it has been suggested by interviewees that a political decision had already been taken to introduce UK-wide breast screening (with support from Health Minister Edwina Currie) and that the Commission was a post-hoc justification exercise. This illustrates the sometimes difficult relationship between policy and subject-matter experts (Weiss, 1977).

There were other significant studies, such as Martin Buxton's 1987 DHSS-commissioned research on the cost-effectiveness of heart transplants but the Rosser index was not used here (see interview above). In these projects and others during the 1980s, such as the Measurement and Valuation of Health (MVH) programme run at York with Alan Williams, Paul Kind and Claire Gudex, economists and medical professionals collaborated with central government and local health authorities to help, as one interviewee put it, to “convert” clinicians and managers to the idea of QALYs (A8). Rather than a ‘breakthrough moment’, the building of research networks was crucial in spreading the idea of QALYs across different environments. Furthermore, medical research grants increasingly required an integral economic component especially for epidemiological studies. Economics was so well entrenched here that one interviewee remarked that “QALYs could have come from epidemiology” (A9).

Within two years of Williams' CABG paper in the BMJ, there was sufficient public awareness of the concept, and concern about its use, that academic health economists found themselves with an opportunity to ‘soften up’ public and political acceptance of QALYs. Newspaper headlines such as ‘Who lives and who dies’, “A game of chance” (*The Times*, 21 December 1987) and “Health care roulette” (*The Guardian*, 5 November 1986) (Ashmore et al., 1989, pp.70–71) were growing. On 16 October 1986, Maynard appeared on Dimbleby's ‘This Week’ on ITV to discuss QALYs with a neonatal intensive care doctor. The same week, he took part in an ITV game show entitled ‘The Life and Death Game’, which used ideas of priority-setting and opportunity costs (Ashmore et al., 1989). In ‘The Heart of the Matter’ on BBC One in October 1986, a fictional health authority was given £200,000 for its population, and had to decide whether “it would get 10 QALYs from dialysis of kidney patients, 266 QALYs from hip-replacement operations or 1197 QALYs from anti-smoking messages” (Harris, 1987).

### 4.3 The politics stream

The third of Kingdon's ‘streams’ relates to politics. During the 1970s, public confidence in the NHS appeared unshakeable but relatively unspoken. Various British politicians, both left and right-wing, have repeatedly emphasised the value and security of the NHS. Nigel Lawson, Conservative Chancellor of the Exchequer (1983–89) called it “the closest thing the English have to a religion” (Lawson, 1992, p.613). Any proposal to introduce further payments in the NHS or to change its financing would be politically risky, as Margaret Thatcher's cautious attitude towards the NHS from 1979 suggested. Policy-makers had to reflect on how decisions around treatments were made. Politics implies negotiation and competition over how society should be organised and ruled, and this here includes negotiations between clinicians and health service managers. Clinical autonomy in decision-making prevailed but was under attack using economics principles – an irony given that clinicians could claim they had always had to make difficult choices in patient care. For economists, it was wrong that a patient was given treatment without active consideration of whether it would benefit them more than another patient, or whether giving them treatment would deprive other potential patients of other treatments (‘opportunity cost’ in economic parlance). Yet this clinical autonomy over patient management was being eroded. As resources became increasingly strained, waiting lists and times for elective care increased, and length of in-patient stays shortened, economists such as Williams pushed for new ideas, suggesting clinical management reforms were required:

[D]octors' specialist skills lie in their ability to diagnose and to know the effects of various courses of action which might then be adopted; and in their ability to implement […] whichever course of action the patient selects. They have no legitimate claim to impose their judgments about the relative valuations of different courses of action upon their patients. (Williams 1985b, p.6; Sheard 2018)

The health economics discourse surrounding QALYs supported this opening-up of clinical management to new ideas. As one government economist interviewed put it: “it is about exposing the consequences of the decisions and the inherent inconsistency that those decisions can lead to” (CS7). An academic economist involved in NHS decision-making noted how QALYs revolutionised the assessment of NHS care:

Alan [Williams] used to describe it so nicely: ‘vertical and horizontal’. People left hospital alive or dead. Literally. The statistics were dead or discharged. And all the medical stuff was about survival and not QoL and so it was a huge step forward. (A1)

One of the first health economists involved in QALY research noted in interview that it “was really uphill work” and that there was significant clinical “resistance to the idea of QALYs” (A10). Another researcher involved in the development of the QALY explained how the York MVH team in the late 1980s worked with “the minority [of clinicians] who were interested in doing this and dared to do it because it was a big thing” (A8). Alliances between economists and specific medical professionals and health authorities to trial QALYs were vital in building support across the three streams and for a policy change.

Budding relationships and tensions between economists and medical professionals are illuminated through a review of the *Lancet* and the *BMJ* for the period 1984 to 1999. Some doctors appeared to be warming to the idea of a QoL-type measurement to allocate health resources, albeit still disputing QALYs *per se*. For instance, David Grimes, a doctor at Blackburn Infirmary, acknowledged in the *Lancet* in 1987 that inefficiencies resulted from rationing being “left to doctors” (Grimes, 1987, p.615). Grimes suggested that QALYs could help make decision-making more open by allowing “lay members of health authorities to decide how to spend their inadequate amounts of money in a way that gives the greatest benefit to society” (Grimes, 1987, p.615.). The epidemiologist Alwyn Smith wrote an article for the Lancet under the title ‘Qualms about QALYs’ (Smith, 1987, p.1135). Although he supported the health economists' view that there were “more potentially beneficial health-care procedures than we have resources to carry out” and a lack of data to inform decision-making, he judged QALYs unfeasible because of the great philosophical and theoretical difficulties that would come from having to decide which patient to treat. According to a researcher involved in developing the QALY alongside clinicians, clinicians underestimated how far-reaching QALYs would become:

[S]ome of the clinicians could have been a bit sceptical but they gave information and were interested in what it could bring. I don't think there was ever … any idea that this was going to be the way that they were going to make decisions. (A8)

It is this budding relationship that eventually led some economists, medical professionals and NHS managers to setting-up what may be termed the precursors of NICE, the Development and Evaluation Committees (DECs) from 1991, in which QALYs were operationalised, bringing together “a group of the health authorities trying to make decisions on what technologies, in the widest sense, to invest in their patch” (Ron Akehurst, witness seminar organised on 27 October 2017 on the development of health economics). Similar groups appeared in the Trent region and later the West Midlands, and a growing number of treatment and technology evaluations became commissioned from economists. Despite these DECs and other similar groups in other parts of the UK, Timmins, Rawlins and Appleby note the limitations of these frameworks in mainstreaming QALY-type evaluations until NICE:

Valuable though their work was, however, it had limited fire power. It remained well short of a nationally authoritative voice recommending what the NHS should or should not adopt, and it did not prevent the controversy over the so-called ‘postcode lottery’. (Timmins et al. 2016, p.30)

### 4.4 Policy entrepreneurs

The previous sections have applied Kingdon's Multiple Stream Analysis (MSA) to demonstrate that in all three areas – problem, policy and politics – there were favourable conditions to support the adoption of the QALY as a new tool within British healthcare policy. MSA also makes possible an assessment of the role of individuals as policy ‘entrepreneurs’ in achieving ‘take-off’ – brokering the transition from emerging idea to full implementation. We here focus on two central actors/groups in the development of the QALY: Alan Williams and the role of the Economic Advisers' Office.

Alan Williams is routinely seen as a pioneer of British health economics, and indeed his arrival at the then-new University of York in 1964 marked the start of an ambitious programme for the new discipline of health economics. Williams embodies the idea of policy entrepreneur, being mentioned by all our interviewees as crucial. One of our interviewees noted that “a lot of it comes down to personalities … Alan was very strong and he had a huge network” (A8). He also appears to have been open to learning about different intellectual environments. He worked at US universities in 1957–1958 and 1963–1964 and undertook a secondment as an economist in the British civil service between 1966 and 1968. This was crucial in enabling him to understand the tortuous process of policy formation. As our interviewees noted, Williams was skilled at translating his and other academics' ideas into a format that policy-makers understood. Although the QALY concept was already in development (through the work of Rosser and Watts, and others), it was when Williams became aware of its potential that he was able to make the concept more palatable, softening it up to make it understandable to a lay and policy audience. Williams was also a well-connected academic who interacted with policy, medical and academic worlds so that he was aware of their demands and values. When political attention shifted to NHS costs and efficiency, economists such as Williams were ready and willing to frame policy solutions in ways that resonated with government interests. For example, in 1986, Williams was one of the founding members of the QoL Measurement Group in partnership with colleagues from Brunel and Middlesex Universities, and which included the government economists Clive Smee and Jeremy Hurst (A3; A8), which further developed QALYs, notably by generating further data and refining the model. This eventually led to the formation of the EuroQoL group in 1993 (Kind, 1998). At the local level, Alan Williams also pushed for the employment of health economists by Regional Health Authorities (RHAs) to facilitate the implementation QALYs (A1; 2; 3).

The Economic Advisers' Office was also a central policy entrepreneur in the development and refinement of QALYs. Government economists David Pole, Jeremy Hurst and later Clive Smee, routinely consulted with academic colleagues and attended conferences, such as the one held at York in 1970 which helped to persuade the government to allocate an initial £20,000 grant to York (CHE Archives, Letter from JD Pole to Jack Wiseman, 3 August 1971) and later HESG meetings. As one DH economist described, the culture within the Economic Advisers' Office during the 1970s-1980s encouraged economists to follow the academic literature (CS1). These economists were critical in the rapid DHSS acceptance of “the idea of cost per QALY” (CS3). They consolidated this by commissioning a number of academic studies on the application of the QALY to build a firm evidence base, a note from Hurst to Smee for instance highlighting how:

As we cover more procedures and continue to improve our estimates, we should gain more confidence in raising questions about the procedures lying at the extremes of the range with a view of limiting the growth of the least cost-effective procedures … (Note from Hurst to Smee, 8 February 1985, DH Burnley, OEA.056.001.009.V002, EAO Study – Cost of Saving Life)

As DH archives demonstrate, there was a significant correspondence between Jeremy Hurst (Economic Adviser) and Clive Smee (Chief Economic Adviser in Health) regarding how QALYs could be implemented (“How much can the NHS afford to spend to save a life or to avoid a severe disability” (ibid.)), the former emphasising that “the only problem will be doing the research to generate the cost-effectiveness data” (Letter from Hurst to Mrs Joan Firth, 12 February 1985, OEA.056.001.009. V002). Clive Smee added that the Economic Advisers' Office “were by then commissioning evaluations all over the place, looking at breast cancer screening” and other areas to build the evidence (Interview).

For academic interviewees who had been involved in the DHSS-funded QoL Measurement Group, they stressed the Department's wish to coordinate the research. For example, one academic recalls being told by a government economist:

He [the government economist] was quite worried that we were undermining the work that they had put a lot of money into with Rachel Rosser and subsequently. (A6)

Another academic researcher emphasised the tight Whitehall management:

[W]e were very closely monitored by the DH […]. There were very defined timelines and you can see from these minutes that they asked us for extra information on part of the study that we provided. There's an agreement that we would disseminate the work. So we had to come with a publication strategy. I think it was partly because it was a lot of money. And also because they really had to show that it had been done properly. It wasn't to fudge around the edges. (A8)

Through these lengthy debates over issues such as which weightings to base the index on, it is possible to see ongoing shifts in ‘coalitions’ between academics and civil servants. These led to significant policy outcomes, such as the decisions that “central tariffs should be based on means not medians, [and] should be [applied to] the whole population and not a sub-group’” (Minute of 1995 QoL measurement steering group). It is unlikely that government economists working in isolation from the academic community would have been able to develop such a sophisticated tool. It required a constant dialogue, through working groups, academic conferences, publications, and commissioning feasibility studies. It also required a broader network between economists and the medical profession, especially the senior medical civil service. For instance, Clive Smee stressed the central role of Chief Medical Officers, especially Ken Calman who “invented the term clinical effectiveness to mean clinical and cost-effectiveness” and “then set up a national screening committee to try and assess using cost per QALY type calculations” (Interview). For Smee:

that was one of the first major clinical decisions that I remember involving … it took for granted that there should be cost effectiveness.

The work of these government economists involved convincing administrators and politicians of the benefits of QALYs:

I was amazed at how quickly the Department accepted cost per QALY and for a long time, we actually kept partly hidden from ministers what we were doing in that area. (Clive Smee, interview)

## 5 Conclusion

This paper has constructed a history of the development and implementation of the QALY concept and associated tools within UK health policy, mobilising Multiple Streams Analysis. We have drawn on previously unexploited archive sources and on semi-structured interviews to highlight how three ‘streams’ – policy, problems and politics – were coupled by ‘policy entrepreneurs’, especially health economists who moved between academia and Whitehall, to exploit the ‘window of opportunity’ that opened in Britain following the 1970s economic crisis. This was only possible because of the creation of, and investment in, a health economics and operational research community, which exploited funding and secondment opportunities. The adoption of QALYs as a usable tool within DH and regional NHS was facilitated by ongoing collaboration between academic and government health economists. Despite apparent tensions over their respective roles, the political pressure to produce a solution to the chronic NHS financial crises enabled an effective working relationship. Although there were no distinct breakthrough points for QALYs as with the creation of NICE in 1999, the various events, networks and individuals we have discussed contributed to the slow build-up and embeddedness of the QALY in the understanding of health and quality-of-life and the formulating of healthcare solutions.

Explaining the various steps and challenges in the formulation of QALYs, and how other options were side-lined, demonstrates that new ideas do not get adopted because they are somehow ‘correct’ or because they ‘speak truth to power’. Rather, through mobilising MSA, we argued that ‘solutions’ are shaped by policy entrepreneurs negotiating ideas in the three streams and pushing for the opening of a policy window to allow change. This historical analysis foregrounds and legitimises the explanatory role of more social factors such as personality and serendipity and provides an alternative to health economists' favoured technocratic explanations of policy adoption in their field. This is important: the QALY concept is still central to healthcare decision-making, especially the role of NICE, but to date there has been little analysis of when and why it gained (and maintained) this authority. Likewise, there has been relatively little discussion of why alternatives such as Disability-Adjusted Life-Years (DALYs) or Healthy-Life Years (HYEs) have failed to gain currency, at least in the UK. At a time when there is renewed discussion over the authority of the pharmaceutical industry, there is a real role for historians to play in supporting civil servants and politicians to understand these choices, and thus to be able to effectively respond to a rapidly changing health policy environment. This paper is a worked example of how to successfully employ MSA alongside history to generate useful lessons for current policymakers. Key findings include: the importance of engaging with appropriate experts (including historians) in the development of new policy at an early stage in the process; and the importance of relations and negotiations, i.e. politics. More research is needed regarding QALYs' international influence and whether such QoL concepts are compatible with local practices and histories.
